# Choosing Wisely: Key Feature Examinations as a Powerful Approach to Foster Clinical Reasoning in Dental Education

**DOI:** 10.1111/eje.70078

**Published:** 2025-11-28

**Authors:** Marc André Ackermann, Tim Becker, Nima Gholamzadeh Biji, Thomas Meyer, Sabine Sennhenn‐Kirchner

**Affiliations:** ^1^ Department of Oral and Maxillofacial Surgery University Medical Center Göttingen Göttingen Germany; ^2^ Division of Medical Education, Study Deanery University Medical Center Göttingen Göttingen Germany; ^3^ Department of Psychosomatic Medicine and Psychotherapy University Medical Center Göttingen Göttingen Germany

**Keywords:** choosing wisely, clinical reasoning, dental education, key feature examination, test‐enhanced learning

## Abstract

**Introduction:**

Clinical reasoning is considered a core skill for physicians, and its training should already be addressed in undergraduate medical education. At the University Medical Center Göttingen, students have been able to engage in formative key feature examinations since 2013. Recent studies have shown that these assessments can improve students' clinical reasoning skills. Such a teaching format was not previously existing in the study of dentistry. This study aims to evaluate the feasibility and efficacy of formative key feature examinations in dental education.

**Materials and Methods:**

In this prospective, randomised, controlled, crossover study, fourth‐year dental students participated in six weekly computer‐based seminars in which complex dental patient cases were presented, and the underlying theoretical knowledge was taught. In alternate weeks, groups of students were invited to either read specially prepared text formats (control condition) or solve key feature cases (intervention) that covered the same theoretical content. Each case contained on average six key feature questions (items) referring to the diagnostic procedure and treatment of the patient presented.

**Results:**

Feedback from the evaluation questionnaire indicated that dental key feature cases were generally well received. In addition, key feature cases can be successfully integrated into dental education in the form of e‐seminars. Learning growth self‐assessment of participants showed a significant improvement in the overall learning objectives. Iterative work with key feature cases was clearly associated with significantly greater improvements in exit and retention test scores compared to text‐based learning.

**Conclusion:**

Repeated formative key feature examinations can be effectively implemented in dental education and dental students' clinical reasoning skills can benefit from working on these cases. In addition, strengthening clinical reasoning skills in undergraduate dental education can help to avoid unnecessary dental interventions in future practice, as outlined in the Choosing Wisely recommendations.

## 1 Introduction

A key gain of undergraduate medical and dental education is to qualify future physicians to adequately take medical histories and choose appropriate diagnostic tests in order to reach correct diagnoses and provide specific treatments [1, 2]. Such complex cognitive processes of gathering information and applying knowledge are referred to as ‘clinical reasoning’. Teaching courses to train clinical reasoning processes should be implemented in both medical and dental education as early as the undergraduate level [3]. Clinical reasoning is an essential prerequisite for making informed decisions in dental practice [4, 5].

In this context, however, medical and dental students should not only learn to make appropriate diagnostic and therapeutic decisions but also to avoid unnecessary or potentially harmful tests and treatments. Therefore, teaching formats aiming to improve the clinical reasoning competence of the students might include the recommendations derived from the Choosing Wisely campaign. This international initiative aims to identify inappropriate overuse of diagnostic and therapeutic procedures that do not provide meaningful benefit for patients or even cause harm [6]. Over the past decade, many medical societies around the world have joined the Choosing Wisely campaign and have formulated specific recommendations for their specialties. So far, only the American Dental Association and Canadian Association of Hospital Dentists have joined the Choosing Wisely campaign. These associations have also identified several unnecessary diagnostic tests and procedures in dental care, and many more are to be expected [7, 8].

One teaching format that has been shown to be effective in developing clinical reasoning skills is case‐based learning, in which students are exposed to clinical problems within exemplary case scenarios [9]. Since case‐based learning with real patients is resource‐intensive and cannot be easily standardized [10], computer‐based learning with virtual patients is a sound alternative: with this, various digital case scenarios can be utilised by numerous students to practise clinical reasoning [11, 12].

The assessment of the learning outcome with regard to clinical reasoning can be performed using so‐called key feature examinations. This test format was developed by Page and Bordage in the 1990s [13], and since then, it has been widely used to assess clinical reasoning skills in health professions education [14]. According to Page et al., a key feature is defined as a critical step in the management of a clinical patient case where students are most likely to make errors [13]. Such critical steps may include reaching correct diagnoses and making appropriate diagnostic and therapeutic decisions, or even avoiding unnecessary or harmful tests and treatments. In key feature examinations, students are confronted with different virtual patient cases and have to solve the clinical problems by answering consecutive questions that focus on the critical steps of the respective case scenarios [13].

However, key feature examinations can be used not only to assess but also to promote clinical reasoning skills. This approach of test‐enhanced learning is based on the findings that repeated retrieval of memorised content (e.g., in the context of formative examinations) stimulates cognitive learning processes and promotes long‐term retention of that retrieved content (so‐called testing effect) [15, 16, 17].

In light of this, a new teaching format was initiated at University Medical Center Göttingen (UMG) in 2013: in the clinical phase of the medical curriculum, students attend weekly computer‐assisted seminars (‘e‐seminars’) in which they complete formative (i.e., non‐graded) key feature examinations referring to the content of the lectures of the respective teaching module. Several studies have shown that this specific teaching format contributes to improving the clinical reasoning skills of medical students [10, 18, 19, 20].

However, while formative key feature examinations have been established in the medical curriculum at the UMG for years, this teaching format has not yet been implemented in the dental curriculum at the same institution. Previous teaching formats at the UMG consisted mainly of lectures, theoretical seminars, model exercises and case presentations in plenary sessions. Digital simulations of complex interdisciplinary cases in the form of key feature cases have not yet found their way into the curriculum of dental education, although there is a need for training in clinical decision making [4, 21]. Even internationally, there seem to be only a few medical schools using key feature cases in dental education: We are aware of only two studies in which key feature examinations were used to assess the clinical reasoning skills of dental students but not to improve them [21, 22]. To date, there is no empirical evidence on how well different educational initiatives, such as key feature cases, influence dental students' clinical reasoning skills [4].

Due to this gap, our working group conducted an initial study on creating dental key feature cases and piloting the cases in dental education [23]. Subsequently, our second study now aims to evaluate the feasibility of integrating key feature cases into dental education in the form of e‐seminars. Furthermore, the efficacy of dental key feature cases in improving clinical reasoning skills will be analysed.

## 2 Methods

### 2.1 Study Design

This project is a randomised, controlled, non‐blinded prospective crossover study in which fourth‐year dental students attended six weekly computer‐based seminars (e‐seminars) in which complex clinical cases in dentistry including theoretical knowledge were taught. The teaching units to be introduced as part of the study focused on piloting e‐seminars designed to improve clinical reasoning skills. During a learning phase of 4 weeks in the summer semester of 2023, two student cohorts were given alternating weekly access to either interdisciplinary dental‐surgical key feature cases (intervention arm) or text modules (control condition) via the Integrated Learning, Information and Work Cooperation System (ILIAS). By using these two learning formats, the study aims to compare test‐enhanced, problem‐focused, case‐based learning and conventional text‐based learning. Regardless of the format used to learn, students were exposed to the same theoretical content each week. E‐seminars were held in our e‐learning resource center.

Only fourth‐year dental students were selected to participate in this study. Before the start of the actual teaching series, the participants were randomly divided into two groups by assigning them a computer‐generated random number (1 or 2) using Excel (Office 365). Furthermore, the students received a comprehensive introduction to ILIAS, the digital realisation of the learning format, and the theoretical basics of key feature cases. While the theoretical content of the case scenarios had already been covered in previous clinical lectures, the students had not yet been exposed to this material in a structured, test‐based or case‐based learning format as implemented in the present study. Examinations and e‐seminars were scheduled to last no more than 45 min, allowing students to complete sessions at their individual pace.

**FIGURE 1:**
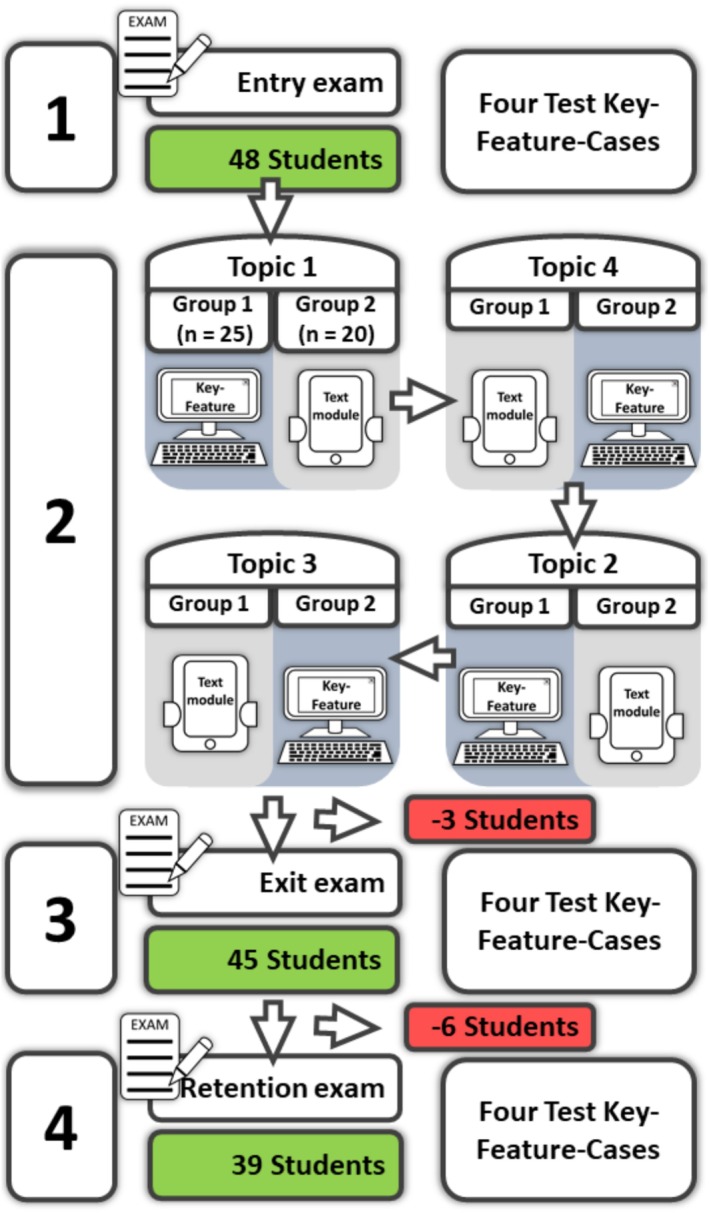
Flowchart of the study procedure.

The teaching series started with a formative entry exam (48 students), which comprised four key feature cases concerning dental‐surgical patient cases (Figure 1). The content of the cases mainly focused on complicated tooth/root fractures, the management of oro‐maxillary sinus perforation after extraction of a maxillary tooth, wisdom tooth removal in the case of pericoronitis and extraction in patients on oral anticoagulant therapy. Each key feature case consisted of a clinical scenario (including the setting as well as the patient's age, gender and symptoms) and up to six consecutive sections (‘serial‐cue approach’ [24]). At the end of each section, students were requested to answer a key feature question that focused on the most appropriate next diagnostic or therapeutic step. These were not multiple‐choice questions (where students could have identified the correct answer from a list of predetermined answer options), but instead required students to actively write their answers in a text box (using at least three letters) and then select an answer from a long pop‐up menu of correct and incorrect answer options [25]. After answering each question, students received automatic feedback with detailed information on the correct answer options as well as the most important incorrect answers.

**FIGURE 2:**
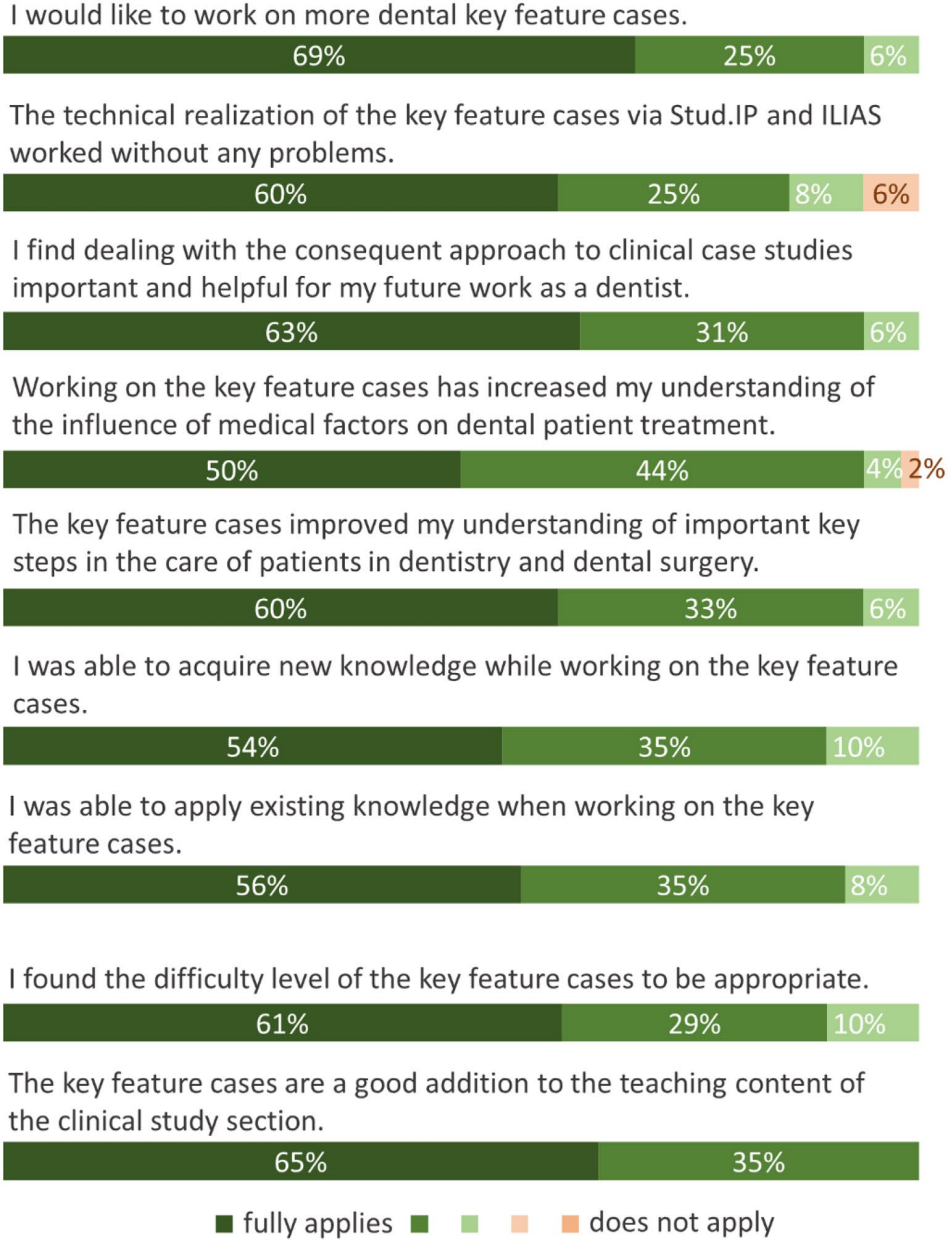
Frequency distributions (in %) of the students' ratings for the nine items of the evaluation questionnaire.

**FIGURE 3:**
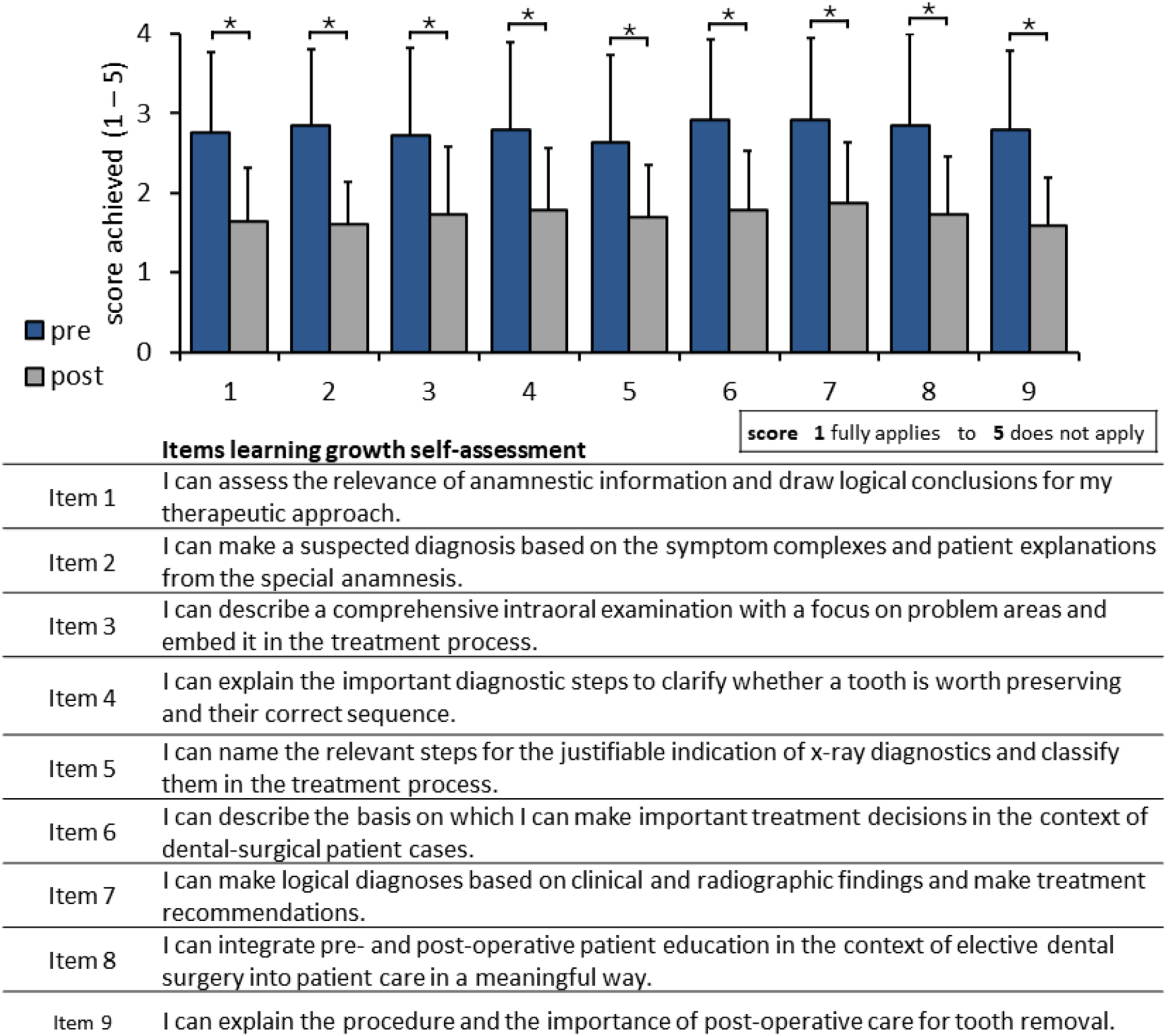
Learning growth self‐assessment during testing for nine defined items. Significant results are marked with an asterisk (*).

**FIGURE 4:**
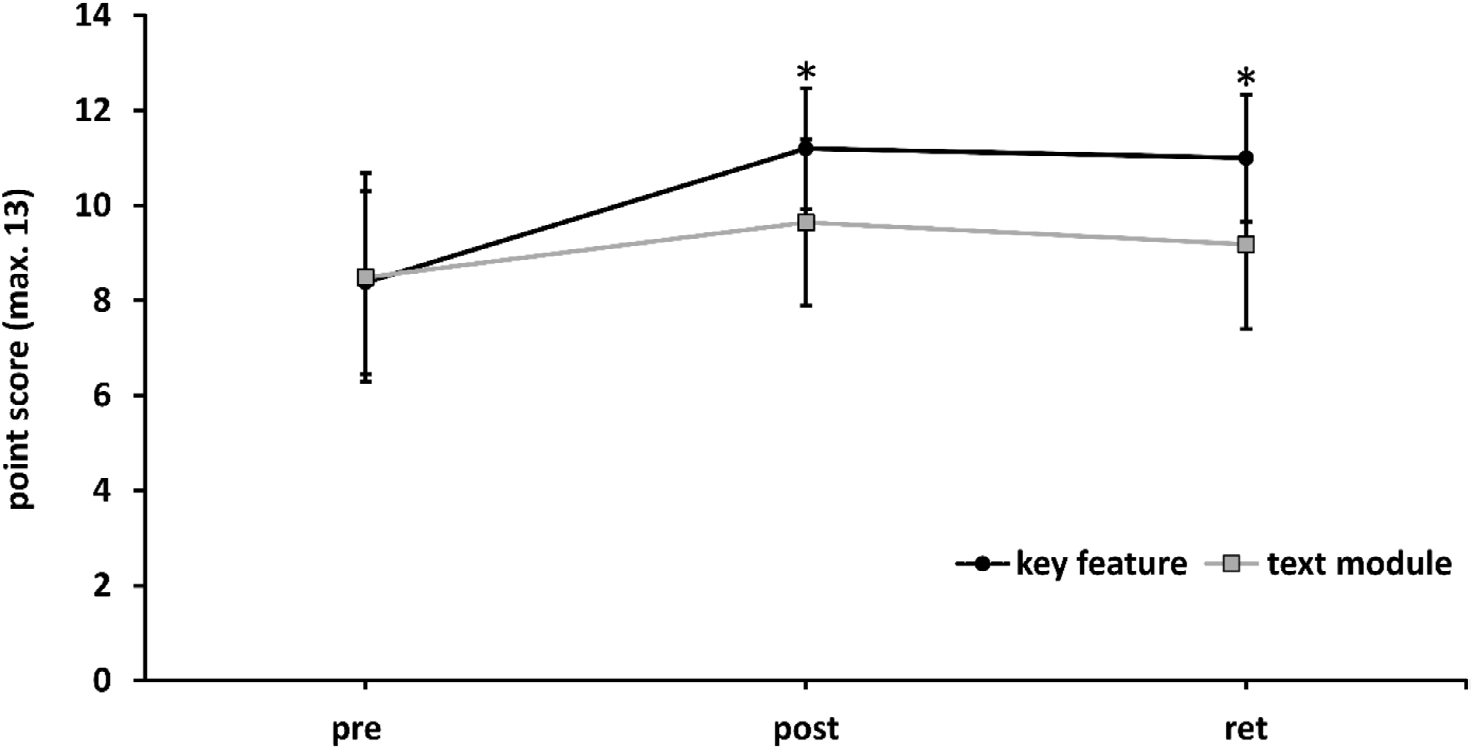
Test scores entry‐, exit‐, retention‐exam for key feature intervention (black) and text control condition groups (grey). Significant results are marked with an asterisk (*).

During the 4‐week learning phase, students alternated each week between two interdisciplinary dental‐surgical key feature cases (intervention) or text modules with the same theoretical content (control condition). Both formats had a comparable duration of approximately 30 min (Figures 1, 2). Each weekly learning unit dealt with one topic of the four key feature cases from the initial examination. After the teaching series, each participant worked through a total of four key feature cases and two text modules, with two key feature cases corresponding to the processing time of one text module. To assess the changes in the students' performance, participants were asked to take a formative final examination, in which they worked on the original four test cases from the first exam (45 students). In addition to the final exam, students were asked to complete a retention exam (39 students) after a 3‐month semester break, which again consisted of the same four key feature cases (Figures 1, 3 and 4). This procedure will form the basis for a later analysis of knowledge retention.

### 2.2 Evaluation

To properly analyse the feasibility and usefulness of integrating key feature cases into dental education, the participants of this study were given the opportunity to conduct an evaluation based on the concept of dental key feature cases. It was also possible to provide free‐text feedback. In addition, the students were able to complete a learning growth self‐assessment on the general learning objectives of the key feature cases [26]. The evaluations and learning growth self‐assessments were saved and analysed using the web‐based evaluation software EVASYS.

### 2.3 Data Analysis

Only students who participated in at least three learning phase e‐seminars were included in the data analysis. For the statistical analysis, the raw data of the results were first processed with Excel (Office 2021) and transferred to SPSS (SPSS Inc. V. 29.0.0.0, Chicago, Illinois, USA). All further statistical analyses were performed with SPSS. The next step in the data analysis was dummy coding to determine semester, gender, and age characteristics of the study cohort. Learning success was then calculated as a difference value (Δ), i.e., the difference between the exit and entry test scores for each key feature case and the overall performance across all four cases. This value was also calculated for the comparison between the retention and entry test. In the following, the differences in performance between the student cohorts are mainly presented as Δ‐values, as described above. Mann–Whitney *U* tests were used to analyse possible differences and their significance between the control and intervention groups, also considering the age, gender and graduate level of the students who participated in the study described above. Furthermore, a linear regression model was calculated with the intention of identifying predictors of student performance. In the following figures, statistically significant results (*p* < 0.05) are indicated by an asterisk (*).

### 2.4 Ethics Committee

Data collection and processing were approved by the ethics committee of the University Medical Center Göttingen (application 15/1/23). The participants were informed about the study and their voluntary participation in the anonymized data use and evaluation of the project before the start of the e‐seminars. All students gave their written consent to participate in this study.

## 3 Results

### 3.1 Characteristics of the Study Cohort

The mean age of the study cohort was 25.8 years and 22% were male. Approximately one‐third (36%) of the participants were in their seventh semester. As shown in Table 1, groups 1 and 2 did not differ significantly in age, distribution of students between the seventh and eighth semesters, or gender composition. Furthermore, there were no significant differences in learning outcomes between group 1 and group 2 for either the interdisciplinary dental‐surgical key feature cases (intervention condition) or the text modules (control condition) (Table 1).

**TABLE 1:** Characteristics of the study cohort including outcome parameters during the testing phase.

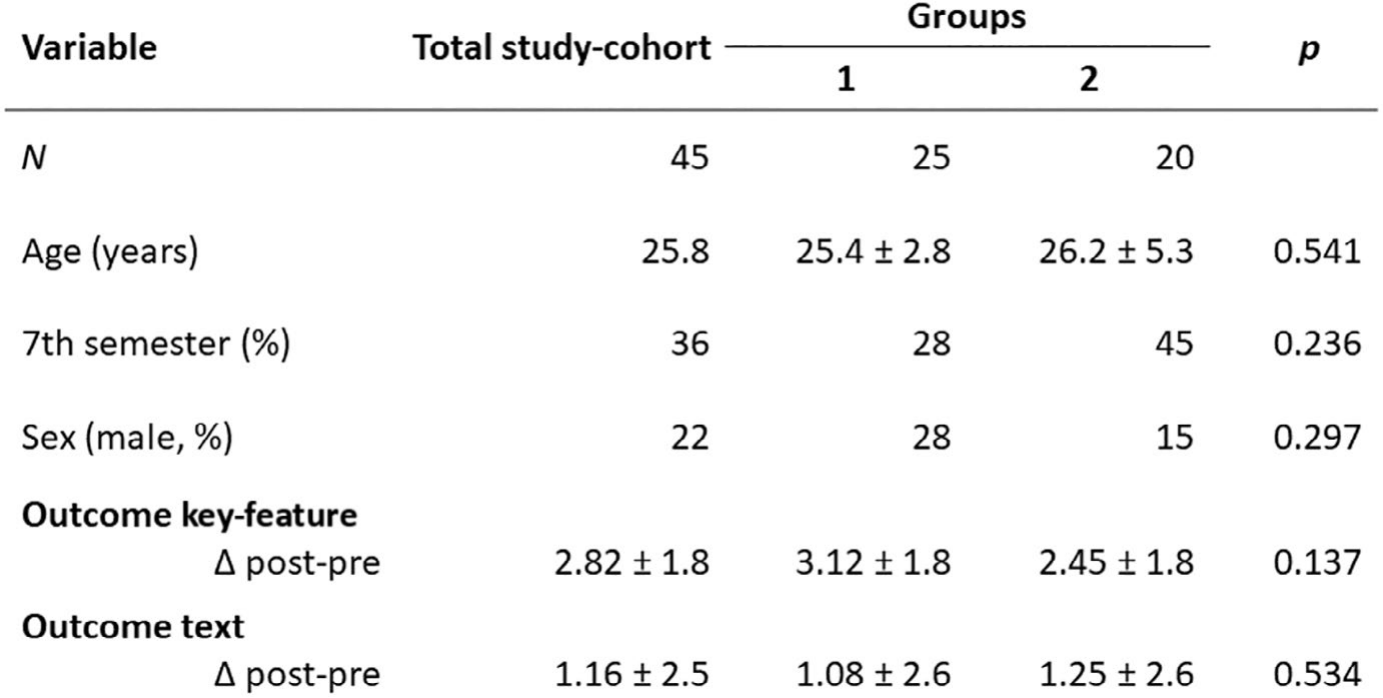

### 3.2 Evaluation Data

The evaluation questionnaire was completed anonymously by all 48 students (100%). The students' assessments of the nine items are shown in Figure 2. In addition to the positive feedback from the evaluation questionnaire, the free‐text comments also indicate that the dental key feature cases were received positively overall (‘I thought the course was great and was able to learn a lot about the process of examinations, diagnoses, etc.’) and that students would welcome an expansion of this teaching format (‘Should be an integral part of the curriculum’). Besides general feedback on satisfaction, the free‐text comments also contain specific suggestions for revising the cases: The majority of the comments refer to the inclusion of more synonymous answer options (e.g., alternative spellings or common abbreviations) as ‘correct’ in the respective long menu answer lists [25] (Figure 2).

### 3.3 Categorised Answers Key Feature Case Three

Forty‐eight students worked on the key feature case ‘Tooth extraction pericoronitis’ as part of the entry exam of the study. The table demonstrates the frequency distributions of correct answers for each question and clusters common incorrect answers into categories. A common incorrect answer to question one was direct radiography at 16.7%. In the second question, the incorrect type of radiography was often selected (33.3%). The most common incorrect answer to the diagnosis question was cyst (37.5%). In addition, students tended to prematurely prescribe antibiotic therapy in questions five (33.3%) and six (12.5%) (Table 2).

**TABLE 2:** Frequency distributions of correct and categorised incorrect answers after the processing of the key feature case ‘Tooth extraction pericoronitis’ by 48 students.

	Percentage (%)	Frequency
Case ‘Tooth extraction pericoronitis’, question 1 (key feature: comprehensive anamnesis)		
Correct answer: anamnesis (+ synonyms)	72.9	35
Wrong answer: clinical examination (+ synonyms)	8.3	4
Wrong answer: x‐ray diagnostics (+ synonyms)	16.7	8
Other incorrect answers	2.1	1
Case ‘Tooth extraction pericoronitis’, question 2 (key feature: x‐ray diagnostics using OPG)		
Correct answer: orthopantomogram (+ synonyms)	64.6	31
Wrong answer: other radiographs (e.g., computer tomography, bitewing view)	33.3	16
Other incorrect answers	2.1	1
Case ‘Tooth extraction pericoronitis’, question 3 (key feature: diagnosis of pericoronitis and indication for tooth extraction)		
Correct answer: pericoronitis (+ synonyms)	41.7	20
Incorrect answer: other inflammations (e.g., pulpitis, periodontitis)	18.8	9
Wrong answer: cyst (+ synonyms)	37.5	18
Other incorrect answers	2.1	1
Case ‘Tooth extraction pericoronitis’, question 4 (key feature: information before tooth extraction)		
Correct answer: preoperative consultation (+ synonyms)	77.1	37
Incorrect answers	22.9	11
Case ‘Tooth extraction pericoronitis’, question 5 (key feature: information on post‐operative rules of conduct)		
Correct answer: consultation about post‐operative rules of conduct (+ synonyms)	52.1	25
Wrong answer: antibiotic therapy (+ synonyms)	33.3	16
Other incorrect answers	14.6	7
Case ‘Tooth extraction pericoronitis’, question 6 (key feature: post‐operative aftercare)		
Correct answer: removal of the sutures (+ synonyms)	37.5	18
Correct answer: control of wound healing (+ synonyms)	29.2	14
Wrong answer: antibiotic therapy (+ synonyms)	12.5	6
Other incorrect answers	20.8	10

### 3.4 Learning Growth Self‐Assessment

The learning growth self‐assessment was completed anonymously by 47 students (98%). It was measured using nine defined items. All nine items showed a significant improvement in clinical reasoning skills on the overall learning objectives (Figure 3).

### 3.5 Test Outcome

A review of the point scores for the entry exam shows that the first student group without intervention scored an average of 8.5 ± 2.2 points and the second student group scored an average of 8.4 ± 1.9 points. The primary endpoint of the exit exam, which took place 6 weeks after the entry exam, demonstrated that students who had practised case topics with key feature cases achieved significantly higher point scores (11.2 ± 1.3 points) than students who had trained with text modules (9.6 ± 1.8 points, *p* < 0.001). Furthermore, the results of the retention test, which followed the final exam after 3 months, were also significantly higher for students who had worked on key feature cases during the learning phase (11 ± 1.3 points) than for those who had worked on text modules (9.2 ± 1.8 points, *p* < 0.001) (Figure 4).

### 3.6 Learning Outcome

**FIGURE 5:**
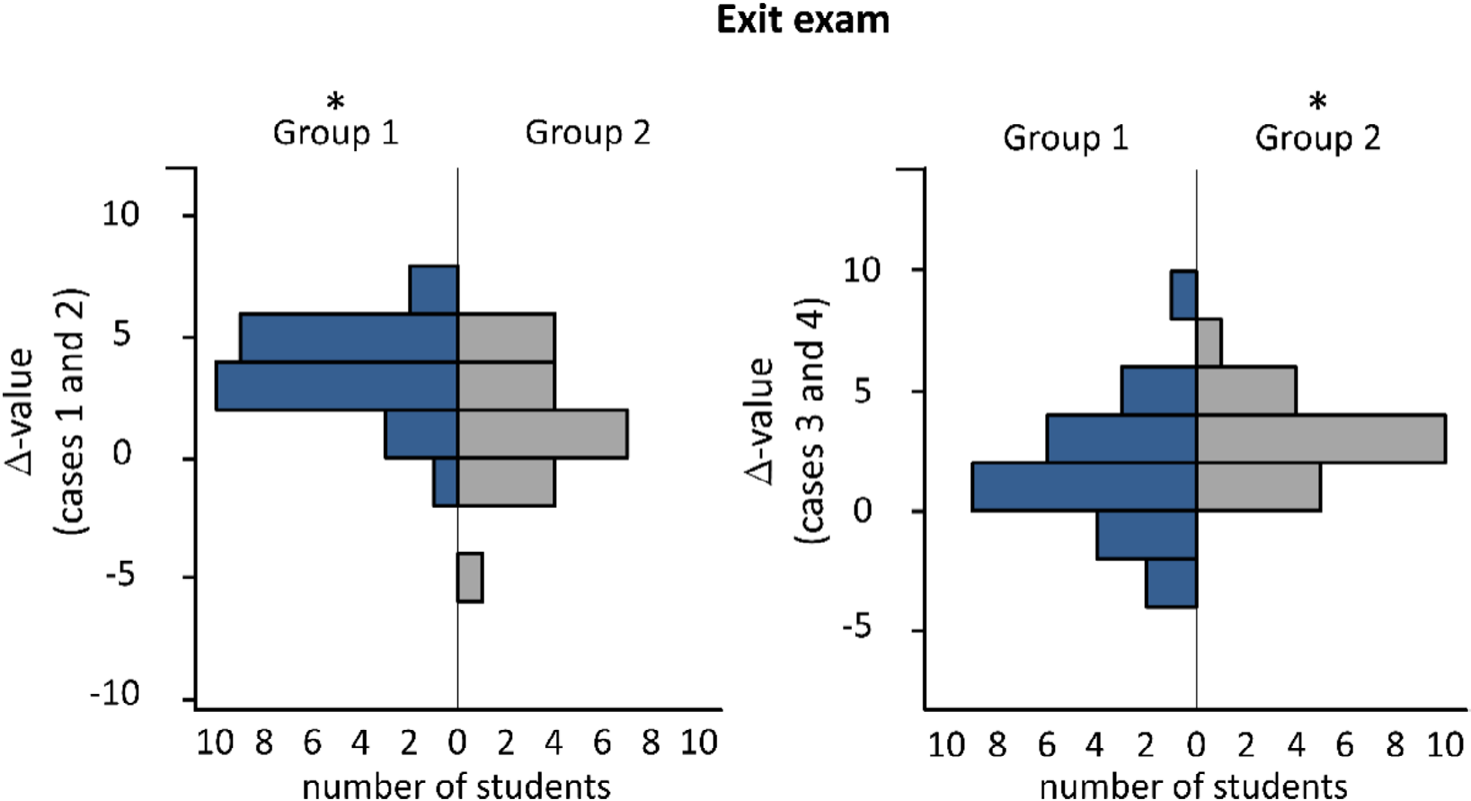
Mann–Whitney *U* test analysis was performed to compare the ∆‑values between the exit examinations of the intervention and control groups. For cases 1 and 2, Group 1 served as the intervention group, whereas for cases 3 and 4, Group 2 acted as the intervention group. Statistically significant results (*p* < 0.05) are indicated by an asterisk (*).

A total of 45 students were included in the cross‐over comparison. In the cross‐over comparison of the two test groups, the Δ‐values of the entry and exit exam comparison were significantly higher for group 1 in key feature cases 1 and 2 (topic complexes trained with key feature cases, median Δ‐value 3, *p* = 0.012) and for group 2 in key feature cases 3 and 4 (topic complexes trained with key feature cases, median Δ‐value 2, *p* = 0.023) than for the respective control group cases (topic complexes trained with text modules) (Figure 5).

**FIGURE 6:**
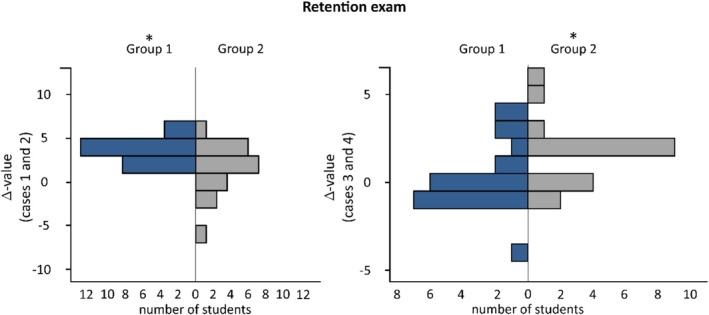
Mann–Whitney *U* test was conducted to compare the Δ‑values from the retention examination between the intervention and control groups. For cases 1 and 2, group 1 functioned as the intervention group, whereas for cases 3 and 4, group 2 served as the intervention group. Statistically significant results (*p* < 0.05) are denoted by an asterisk (*).

A total of 39 students completed the retention exam and were included. In the cross‐over comparison of the two test groups, the Δ‐values of the entrance and retention exam comparison were significantly higher for group 1 in key feature cases 1 and 2 (topic complexes trained with key feature cases, median Δ‐value 3, *p* = 0.007) and for group 2 in key feature cases 3 and 4 (topic complexes trained with key feature cases, median Δ‐value 2, *p* = 0.04) than for the respective control group cases (topic complexes trained with text modules) (Figure 6).

In a linear regression model, age was found to be a positive predictor for learning outcome in the key feature examinations (*beta*‐coefficient = 0.366, *p* = 0.015). However, neither semester nor gender was associated with test outcome.

## 4 Discussion

In this study focusing on the implementation of key feature cases in dental education, two important objectives were achieved. Firstly, the feasibility of integrating key feature cases into dental education was experimentally verified. Additionally, the study groups' test results demonstrated the efficacy of dental key feature cases in increasing clinical reasoning skills and through these clarifying misconceptions about treatment procedures in common dental‐surgical patient cases.

To identify any biases due to differences in gender, age, semester of study or previous clinical reasoning skills, an analysis of the characteristics of the cohort was performed. Study groups 1 and 2 showed no statistically significant differences in age, distribution of students between the seventh and eighth semesters, or gender composition. Furthermore, there were no significant differences in learning outcomes between the two groups for either the key feature cases or the text modules (Table 1). These results suggest that both cohorts were comparable in terms of their learning performance, thus ensuring a balanced basis for the evaluation of the intervention effects. It can be assumed that the learning outcomes are primarily related to the training interventions and not to biases caused by specific cohort characteristics.

The incorporation of key feature cases into the e‐seminars was generally perceived positively by the participating students. In the free‐text comments, the participants expressed a desire for further key feature cases, as these are seen as a good opportunity to apply and deepen acquired knowledge in specific clinical contexts. The completed evaluation questionnaires also underline that the participants in this study appreciate the focus on important key steps in dental patient care and the influence of interdisciplinary medical factors on the treatment process in dental casuistry. Choosing the appropriate steps and considering cross‐disciplinary medical factors are seen as crucial for their future dental practice. In addition, most students stated that the technical implementation of the key feature cases via Stud.IP and ILIAS worked without any problems (Figure 2). This leads to the conclusion that the integration of dental key feature cases in the form of e‐seminars is basically a feasible approach. However, it should be noted that most of the free‐text comments contained concrete suggestions for revising the cases in the form of more synonymous answer options [23]. The inclusion of long menu questions clearly increases the amount of work required to regularly revise the correct answer options [7]. In line with these evaluation data, the learning growth self‐assessment on the overall learning objectives of the key feature cases indicates that the participants assess their clinical reasoning skills in terms of the defined items as significantly improved after training with key feature cases (Figure 3) [26].

While the overall feasibility and acceptability of formative key feature examinations were confirmed by student evaluations, it should be noted that the successful implementation of such formats in other institutions may depend on several factors. These include local access to appropriate digital platforms such as ILIAS, existing faculty expertise in developing clinically relevant key feature cases, and sufficient institutional support. As described in a previous publication [23], the process of developing, technically integrating, and revising cases can be time‐consuming and requires a clearly defined workflow. These experiences highlight the value of a standardised development framework for key feature cases, which may help other departments ensure high initial quality of new items even before piloting, thereby reducing the need for extensive revisions in later implementation phases [23, 25].

The test results, in complement to the evaluation and learning growth self‐assessment data, confirm the positive effect of working with key feature cases on the clinical reasoning skills of dental students. In general, preparation for the exit and retention exam with key feature cases was clearly associated with significantly higher scores in the exams than training with text modules (Figure 4). Moreover, the cross‐over comparison between the two test groups showed that the ∆‐values, indicating individual learning success, were significantly higher for the groups that trained on topic complexes with key feature cases than for their respective control groups, both in the entry and exit test and in the entry and retention test comparison (Figures 5 and 6).

On average, students scored just over 65% on each key feature (Figure 4), with no significant difference between the scores of seventh and eighth semester students. Assuming that students in the eighth semester should have accumulated a full semester of additional procedural clinical knowledge, it is reasonable to expect that students in the eighth semester would, on average, score higher on the entry exam. However, the entry exam scores were comparatively low, indicating a neglect of clinical reasoning skills in previous teaching [13, 14]. As an example, case three of the entry exam highlights the fact that there are gaps in knowledge or misconceptions, e.g., regarding the correct procedure for indicating X‐ray diagnostics and the use of antibiotics in the course of treatment for conventional wisdom tooth removals (Table 2). By using key feature examinations as a teaching intervention, it is possible to specifically address misconceptions in clinical treatment procedures as outlined in Choosing Wisely recommendations [7, 8]. This might be a good chance to reduce the inappropriate overuse of diagnostic and therapeutic procedures in future dental practice.

Within medical education, the integration of test‑, problem‑, and case‑based learning methodologies in the form of key feature cases is recognised as a successful approach, as several studies have shown improved students’ clinical reasoning skills [10, 18, 19, 20]. Such improvements are presumably achieved by reducing a clinical problem to its critical decision points (key features), which helps learners to identify and prioritise relevant clinical cues and patterns, thereby building mental representations (or ‘disease scripts’) of common patient problems [3, 5, 24]. Providing immediate feedback on decisions in key feature cases can help students to review their reasoning and promote metacognitive skills (i.e., reflecting on their own thinking) [20].

Despite these promising cognitive mechanisms stimulated by key feature examinations, this concept has not yet been widely adopted in dental education. The present study is one of the first to use this approach in dental education with the aim of improving students' performance in clinical reasoning. The questions used in the learning phase and formative examinations targeted very common or important dental surgical problems, and all questions were piloted [23].

The application of randomisation, the utilisation of a cross‐over study design, and the comparative analysis of cohort characteristics collectively served to minimise the risk of bias. However, it should be noted that the non‐blinded study design could be a potential source of bias. As the students were aware of the format of their respective learning intervention, expectations or motivational effects cannot be completely avoided. While the objective nature of the test format and the use of standardised instructions should minimise such influences, the possibility remains that students in the key feature group were more engaged due to the interactive format. Future studies could introduce blinding at the level of intervention labelling or include objective measures of engagement to avoid this potential bias. The results of this study cannot be generalised due to its monocentric nature and the missing test of actual student performance in a clinical setting. Therefore, this study cannot establish a conclusive causal relationship between repeated case‐ and problem‐based testing and a more favourable patient outcome. Larsen et al. demonstrated that test‐enhanced learning using both written tests and standardised patients led to improved clinical application of knowledge, suggesting a potential link between repeated testing and improved patient‐related outcomes [27]. Future research should investigate this relationship by incorporating direct assessments, such as resource‐intensive objective structured clinical examinations (OSCEs) or bedside teaching. Although the key feature cases used in this study were interdisciplinary in nature, only four case structures were used, limiting the transferability of the findings to other areas of dental education. Multi‐center and longitudinal studies are needed to further investigate the external validity and long‐term impact of such interventions on clinical performance and patient care.

## 5 Conclusion

While we are aware of two previous studies that used key feature examinations to assess clinical reasoning skills and revealed that clinical reasoning skills were insufficiently developed even in senior semesters [21, 22], the present study differs in its purpose and design. Rather than focusing exclusively on assessment, we implemented interdisciplinary dental key feature cases to actively improve clinical reasoning skills. To our knowledge, this is the first study to evaluate the efficacy of such an intervention in dental education. Thus, our findings contribute to the existing body of knowledge by extending the use of key feature cases from assessment tools to structured educational interventions. Training with key feature cases is specifically designed to address clinical reasoning problems, and iterative work with this learning format was clearly associated with significantly greater improvements in final and retention test scores compared to text‐based learning. Considering all the analysed data, we are strongly led to the conclusion that repeated formative key feature examinations can be effectively implemented in dental education in the form of e‐seminars and that dental students' clinical reasoning skills can benefit from working on complex patient case simulations. Furthermore, strengthening clinical reasoning skills by introducing key feature cases in undergraduate dental education might help to prevent unnecessary interventions in the future work life of practitioners, as outlined in the Choosing Wisely recommendations.

## Author Contributions

Dr. Marc André Ackermann: Project conception and management, creation of the key feature cases, communication with experts, writing the ethics application, communication with students, teaching in e‐seminars, data collection, data analysis, manuscript writing and editing. Tim Becker, MA, MME: Support with the project concept, didactic advice on the creation of the key feature cases, technical implementation in the learning platform, data analysis, writing the manuscript. Nima Gholamzadeh Biji: Support with the project concept, professional advice on the creation of the key feature cases, data analysis, manuscript writing and editing. Prof. Dr. mult. Thomas Meyer: Support with the project concept, professional advice on the data collection and analysis, data analysis, revision of the manuscript. PD Dr. Sabine Sennhenn‐Kirchner, MME: Project idea, conception and management, validation of the key feature cases, communication with experts, support for the seminars, revision of the ethics application, revision of the manuscript. All authors read and agreed to the final version of the manuscript.

## Funding

Our project was funded by the Department of Oral and Maxillofacial Surgery, University Medical Center Göttingen, and by study quality resources of the University Medical Center Göttingen.

## Conflicts of Interest

The authors declare no conflicts of interest.

## Data Availability

The data that support the findings of this study are available on request from the corresponding author. The data are not publicly available due to privacy or ethical restrictions.
